# Forgotten DJ Stent with a Large Calculus at Its Distal End in an Ileal Conduit Diversion

**DOI:** 10.1155/2014/684651

**Published:** 2014-08-18

**Authors:** Anurag Puri, Vinod Priyadarshi, Nivedita Raizada, Dilip Kumar Pal

**Affiliations:** Department of Urology, IPGMER and SSKM Hospital, Kolkata 700020, India

## Abstract

Calculus formation in an ileal conduit following cystectomy is a known complication. Encrustation and formation of calculus may also occur over a DJ stent retained for a long period; but this is never reported in patients with conduit diversion because of close surveillance of these patients. Here we report first case of a large calculus encrusted over a forgotten DJ stent within an ileal conduit in a man who had undergone urinary diversion following radical cystectomy for carcinoma urinary bladder 8 years earlier.

## 1. Introduction

Ileal conduit is the most commonly used urinary diversion method after radical cystectomy [1]. Stone formation in such conduit is a known long term complication [1, 2]. A prolonged in situ presence of a stent may also lead to encrustation and stone formation [3]; however, it has never been reported in this group of patients with conduit who usually remain under close surveillance.

## 2. Case Report

**Figure 1:**
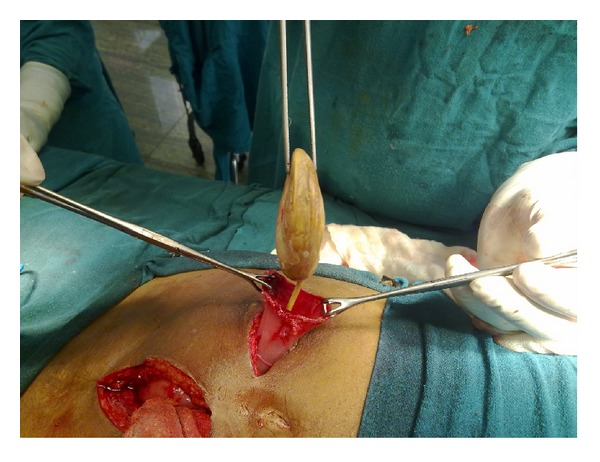
Intraoperative photographs.

A 52-year-old man with complaints of intermittent left flank pain for the last 6 months was referred to our urology Outpatient department (OPD). The patient had history of urinary bladder carcinoma for which he had undergone radical cystectomy with ileal conduit diversion 8 years back at some other hospital, after which the patient was lost to followup at that urological centre. In between these years, besides his recently developed left flank pain, there was no history of any major illness, haematuria, calciuria, or febrile UTI. Clinical examination was essentially normal, showing a well-functioning urostomy and a healthy surgical scar, except for a vague, nontender lump felt in the left lumbar region. His blood counts as well as serum creatinine and electrolytes were normal. Urine analysis did not suggest any malignant cell but there was evidence of pyuria, though the culture was sterile. A CECT whole abdomen was obtained that ruled out any recurrence of the malignancy. It however showed a stent in ileal conduit, along with a large nonobstructing calculus at its distal end. The proximal end of the stent was free. To remove the stone and stent, an initial local exploration of the stoma was done which included freeing of the stoma from the skin edges and attempt at extraction of the stent. This did not meet with success. A separate midline incision was then made to push the stone from behind and guide it out of the conduit along with the stent (Figure 1). After delivery of stone along with the stent, stoma was refashioned and endoscopic examination was performed. No other abnormality was found in the conduit and in either ureter. Postoperative period was uneventful.

**Figure 2:**
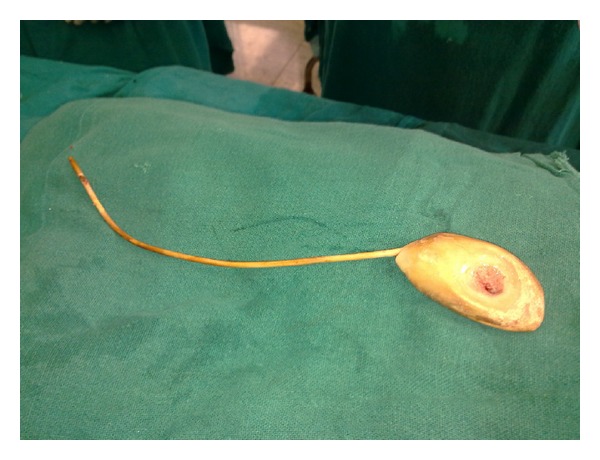
Intraoperative. Removed DJ stent with the calculus at its distal end.

The stone measured was 3 cm × 6 cm size and it was struvite in nature (Figure 2).

Patient was briefed about his disease and counselled for a strict follow-up.

## 3. Discussion

Urinary diversion is an essential part in the management of patients who undergo extirpative surgery of the bladder like radical cystectomy [3]. Even in this current era of continent urinary diversion and neobladder formation, ileal conduit is still considered a safe procedure and the gold standard to which newer forms of urinary diversion are compared. Being a low pressure reservoir as long as outflow is unobstructed, which requires the shortest length of bowel and exposes the mucosa to urine for a shorter time, ileal conduit has lesser complication than continent diversions [1]. Still, an ileal conduit is blemished with its own set of complications. The various complications manifest at different times, and these patients require close surveillance for even decades after the urinary diversion [2]. One of the long term complications noted after urinary diversion is urolithiasis and the incidence varies from 4.9% to 15.3% [3]. The majority of these stones are composed of calcium, magnesium, and ammonium phosphate and occur mostly as a result of metabolic acidosis induced hypercalciuria, stomal stenosis, structural and neuromechanial alteration induced urinary stasis, and urinary tract infection with urea splitting organism or due to presence of foreign bodies such as stapler or nonabsorbable suture [1, 3]. They could be either in upper tract or in conduit itself. Upper urinary tract calculi in such patients usually have a symptomatic presentation while calculi located within the urinary diversion can have varied presentations, for example, incontinence, retention, catheterization difficulties, hematuria, abdominal pain, and recurrent or persistent urinary tract infection. In many cases they remain asymptomatic and their diagnosis requires a high index of suspicion [3].

A ureteral stent is often used to support ureterointestinal anastomosis in such urinary diversions and to drain the ipsilateral renal unit. Although the quality and durability of double J stents has improved in recent years, encrustation of these urinary stents still remains a constant threat. Incidence as well as magnitude of stent encrustation increases as indwelling duration prolongs [4, 5] and hence stone burden is usually large with forgotten stents. Silicone stent is found with significantly less incidence of encrustation than polyurethane stents; however, extension of indwelling time eliminates this difference [5]. The deposition of encrusted material on retained ureteral stents can occur in both infected and sterile urine. In infected urine, organic components in the urine crystallize out onto the surface of biomaterial and become incorporated into a bacterial biofilm layer. Urease produced by the adhered bacteria hydrolyses the urea to produce ammonia that elevates urinary pH, favoring the precipitation of magnesium and calcium as struvite and hydroxyl apatite. Although the exact mechanism of encrustation in sterile urine is unclear, it appears to be dependent on the pH, ionic strength, and biomaterial hydrophobic properties [4]. Forgotten ureteral stents are observed in urologic practice because of poor compliance of the patient or failure of the physician to adequately counsel the patient as in the present case [4, 5]. Most surgeons will leave a single J stent with the distal end externalized out of the stoma. This would make it less likely for the patient to be lost to followup with a retained stent.

A patient of urinary bladder carcinoma who underwent radical cystectomy and ileal conduit requires sincere long term surveillance for tumor recurrence and high treatment related morbidity and complications. Slaton et al. [6] recommended stage specific surveillance protocol that suggests annual screening with physical examination, serum chemistries, and chest radiograph for patients with pT1 disease, semiannual evaluation for patients with pT2 disease, and quarterly evaluation for patients with pT3 disease along with semiannual CT scan. Considering the long term risk of stone formation in these conduits, Patel and Bellman [7] recommended annual KUB X-ray and flexible lower-tract endoscopy to look for urolithiasis. In doubtful cases, helical CT may be useful [5, 8]. The same is expected in patients with indwelling stent that should be monitored while in place with appropriate imaging such as X-ray KUB at regular intervals, promptly removed when no longer needed, and changed periodically if chronically indwelled [4, 5]. Forgotten stents can be managed with variety of modalities such as ureteroscopic removal of the stent when placed in ureters. For stents in the conduits, laparoscopic as well as open surgical intervention can be used. A stone in conduit requires a more meticulous approach and should be conservative or minimally invasive, surgery being reserved for those cases in which stone extraction is not safe using other methods [8]. In the present case open extraction of the stone and stent was done due to the large stone burden. To the best of our knowledge, a forgotten double J stent with such a large calculus at its distal end in an ileal conduit has not been previously reported in English literature. In this era of surveillance, missing a DJ stent in a patient of urinary bladder cancer who had undergone radical cystectomy and ileal conduit is highly unusual and it is a failure on part of caregiver as well as of patient who defaulted on the followups and presented after eight years without any major morbidity in between. By means of this case report, we wish to emphasize on the significance of the regular and stringent followup of such patients with urinary diversion on long term basis.
